# Biomechanical homeostasis in ocular diseases: A mini-review

**DOI:** 10.3389/fpubh.2023.1106728

**Published:** 2023-01-17

**Authors:** Ying Cheng, Tianmin Ren, Ningli Wang

**Affiliations:** ^1^Beijing Ophthalmology and Visual Sciences Key Laboratory, Beijing Tongren Eye Center, Beijing Institute of Ophthalmology, Beijing Tongren Hospital, Capital Medical University, Beijing, China; ^2^Collaborative Innovation Center for Brain Disorders, Beijing Institute of Brain Disorders, Capital Medical University, Beijing, China; ^3^Beijing Key Laboratory of Fundamental Research on Biomechanics in Clinical Application, Capital Medical University, Beijing, China

**Keywords:** biomechanical homeostasis, keratoconus, glaucoma, diabetic retinopathy, myopia

## Abstract

Diabetes mellitus-induced hyperglycemia is responsible for multiple pathological ocular alternations from vasculopathy to biomechanical dyshomeostasis. Biomechanical homeostasis is crucial to maintain the normal physiological condition of the eyes. Biomechanical features vary in eye tissues regarding different anatomical positions, tissue components, and cellular functions. The disturbance in biomechanical homeostasis may result in different ocular diseases. In this review, we provide a preliminary sketch of the latest evidence on the mechano-environment of the eyeball and its possible influencing factors, thereby underscoring the relationship between the dyshomeostasis of ocular biomechanics and common eye diseases (e.g., diabetic retinopathy, keratoconus, glaucoma, spaceflight-associated neuro-ocular syndrome, retinal vein occlusion and myopia, etc.). Together with the reported evidence, we further discuss and postulate the potential role of biomechanical homeostasis in ophthalmic pathology. Some latest strategies to investigate the biomechanical properties in ocular diseases help unveil the pathological changes at multiple scales, offering references for making new diagnostic and treatment strategies targeting mechanobiology.

## Introduction

Diabetes mellitus (DM) imposes a heavy economic burden worldwide with a detrimental impact on ocular health. Chronic exposure to hyperglycemia exerts toxicity to cells and aggravates the metabolic dysfunction in ocular tissues at both physiological and pathophysiological scales. Glucose-rich ambiance is the major culprit of DM-related eye diseases, which could stimulate the polyol pathway, boost the production of advanced glycation end-products (AGEs), activate protein kinase C, increase oxidative stress, and upsurge inflammatory pathways (1). For instance, hyperglycemic conditions promote the activity of aldose reductase in the polyol pathway and induce chronic accumulation of sorbitol in the lens, which further raises the osmotic pressure along with the excessive oxidative stress, and eventually contributes to the onset of cataracts (2). Meanwhile, DM-induced hyperglycemia could trigger subsequent ocular changes that range from the impairment of vascular supply to the elevation of intraocular pressure (IOP). As reported, for every 10 mg/dL increase in fasting serum glucose, IOP increases by 0.09 mmHg in men and 0.11 mmHg in women (3). The IOP level was found to be lower in DM patients with adequate control of the blood sugar than in those without (4). Moreover, owing to the end-organ effect of uncontrolled glucose levels, DM has also been considered as a potential risk factor for other deleterious abnormalities such as glaucoma (5). Therefore, maintaining biomechanical homeostasis is vital for eye health in the context of oculopathy management including DM and glaucoma.

**Figure 1:**
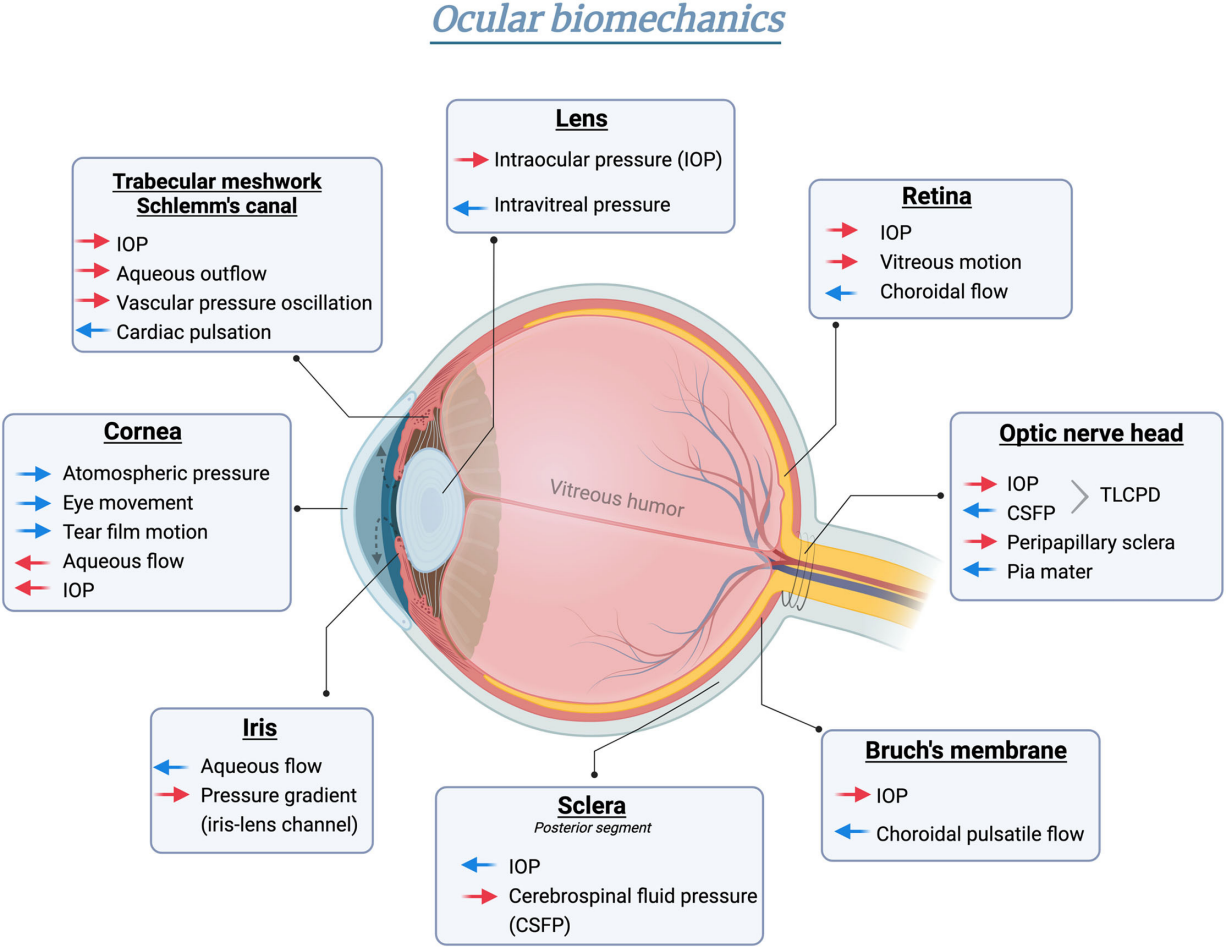
Schematic illustration of biomechanical homeostasis in different ocular tissues. Red arrows indicate intrinsic stress formed in the globe, whereas blue arrows represent the counterforce or “external” pressure outside the eye. Created in BioRender.com.

As the only visual sensation organ, the eye is physiologically subjected to multiple sources of pressure, which is referred to as ocular biomechanics (Figure 1). The term “biomechanics” defines the physical responses of biological tissues under different pressure influences (6). Starting with the anterior compartment, the cornea is the outermost component of the eye globe. Exposed to the open air, the cornea directly bears the exterior stimuli generated by atmospheric pressure (7), eye movement (8), and tear film motion (9), etc. To counterbalance, the internal stresses were fostered by aqueous humor (10) and IOP (7). As the continuous tissue of the cornea, the anterior sclera also percepts comparable stresses and strains (11). However, the posterior sclera [peripapillary sclera and scleral canal connected to lamina cribrosa (LC)] is mainly affected by “external” stresses imparted by cerebrospinal fluid pressure (CSFP) from the back of the eye globe (12). Regarding the anterior chamber angle, the aqueous outflow is driven by the mechanical strain on the trabecular meshwork (TM) and the shear stress arising from the circumferential flow through the Schlemm's canal (SC), thereby modulating the IOP homeostasis (13). Additionally, SC and TM are also subjected to the ocular pulse generated from either cardiac pulsation in the retina and choroid or the pressure oscillations in the episcleral vessels (14). The lens capsule completely encloses the crystalline lens, with its thicker and more durable anterior capsule (toward aqueous humor) accommodating to the IOP (15), and the posterior capsule (toward vitreous cavity) facing the intravitreal pressure (16). The viscosity and elasticity properties of the capsule membrane exhibit high resistance to extrinsic mechanical strength and intrinsic deformative stress occurring in the lens shape alterations (16). Under physiological status, the anterior and posterior chamber is interconnected by the iris-lens channel allowing the aqueous flow. The pressure difference between these two chambers is relatively equilibrated, with only <1 mmHg difference in human eyes estimated by a mathematical model (17).

The posterior chamber of the eye is a spherical cavity filled with gel-like vitreous humor buffering the mechanical stimuli exerted on the lens or retina under both static and dynamic nature (18, 19). Specifically, the vitreous chamber serves as a torsionally oscillating sphere in the eye rotation process. The subsequent vitreous motion would result in a small shear stress on the retina in a radius manner (20). Bruch's membrane (BM) is a thin acellular lamina at the inner layer of the choroid, subjected to constant pressure-induced mechanical stress resulting from the choroidal flow changes resonating cardiac pulsation (21). As the physical and biochemical barrier between the retinal pigment epithelium and the choroid, BM facilitates metabolic transportation across tissues *via* the stress-strain (22). The biomechanics at the optic nerve head (ONH) of the posterior orbit are regulated in a more complex way. Anatomically, the eye and the brain are connected by the optic nerve passing through the translaminar region and the subarachnoid space. Biochemically, the translaminar cribrosa pressure difference (TLCPD), formed by IOP and intracranial pressure (ICP) across the ONH, establishes significant levels of pressure gradient along the nerve tract (23). Meanwhile, other properties, including orbital tissues and pia mater, are also involved in the mechanical features imposed on the optic nerve (12). Taken together, these features delicately manifest the regional specialization of ocular biomechanical dynamics.

Under normal physiological conditions, the ocular biomechanics is generally kept in a dynamic-balanced fashion, with temporary fluctuations in stresses and strains. As the predominant and solely modifiable risk factor in glaucoma, IOP has a clear circadian oscillation pattern. Thus, the 24-h IOP recording is better at reflecting the biological features of IOP (24). The normal IOP, lying between a range of 10–21 mmHg, can be termed as “normal resting IOP.” The normal resting IOP is influenced by multiple extrinsic factors. Specifically, the IOP level alters with eye movement, generally increasing in the eye upgaze phase, and decreasing in the inferonasal gaze phase (25). Weekly and seasonal variations of IOP are also observed (26). Notably, eyelid-related maneuvers such as eyelid squeezing or rubbing can trigger a transient IOP spike excessing normal range on a time scale of less than a second (27). Here, we defined this specific type of IOP elevation as “transient IOP fluctuation.” Under normal conditions, these transient “attacks” would not lead to any observable functional or structural damage. One possible speculation is that these short-term IOP spikes are managed by some mechanical response units which can help neutralize and prevent the potential damage caused by pressure insults.

At the cell level, the mechanical response unit mainly refers to the mechanosensitive channels, categorized into Na^+^-permeable, K^+^-permeable, and non-selective cation (TRP, Piezo) channel families. These channels serve as bandpass filters allowing transmission of certain types of mechanical loading pressure such as tension, stretch, shear flow, and compression at specific amplitude. Previous studies identified the expression of Piezo and TRP family channels in the cornea (28), TM (29), and retina (30), which exerts a vital impact on the regulation of inflammation, oxidative stress, cell apoptosis, and neurotransmission, etc. (31). Widely distributed mechanosensitive channels serve as the multi-functional mechanical transducer and play parts in maintaining ocular biomechanics. The disruption of mechanical homeostasis initiated by the dysfunction of mechanosensitive channels or other pathological stimuli may exacerbate the damage to the stressed tissues, thus leading to the occurrence and progression of ocular diseases.

## Disorder of biomechanical homeostasis in ocular diseases

### Diabetic retinopathy

Diabetic retinopathy (DR) is one of the most prevalent complications of DM. The progression of DR is associated with chronic DM status, hyperglycemia, hypertension, dyslipidemia, higher body mass index, and smoking (32). Recent studies demonstrated the potential association between glaucoma and DR, as they share several common risk factors (e.g., blood pressure, obesity, serum total cholesterol, etc.) and pathophysiological features (e.g., impairment of vascular supply, and neuroretina degeneration, etc.) (33, 34). We previously confirmed that the body mass index (BMI) is positively correlated with CSFP (35), and the latest meta-analysis demonstrated that obesity (BMI > 30 kg/m^2^) was a risk factor for non-proliferative DR. Collectively, we speculated that the high BMI induces the elevation of CSFP, which may lead to the dysfunction of capillary reflux and the upregulation of retinal venous pressure. Retinal venous pressure is reported to be increased in both DR and glaucoma (36). The elevated retinal venous pressure causes hypoxia and tissue edema, resulting in potential pathologic changes including microaneurysm and cotton wool spots at the early stage of DR. Moreover, the increased retinal venous pressure may trigger mechanosensitive channels such as TRPV4 in endothelial cells. TRPV4 activation is linked to higher BRB permeability, and the genetic ablation of TRPV4 could efficiently alleviate retinal edema and BRB compromise in diabetic mice (37, 38). Thus, targeting mechanosensitive channels like TRPV4 could be a promising therapeutic strategy for the treatment of DR.

Intriguingly, the possible association between DM and keratoconus (KC) was also reported. McKay et al. (39) proposed a similar collagen crosslinking mechanism in the development of both diseases, they hypothesized that DM is associated with increased ACEs that led to inter- and intramolecular crosslinking, thus increasing the corneal rigidity. On the contrary, KC is characterized by decreased mechanical stiffness and secondary corneal ectasia. Hence, excessive crosslinking in DM may protect against KC development, but further studies are required to verify this hypothesis.

### Keratoconus

The cornea is the outermost transparent tissue of the eye, its biomechanical properties, such as strength and stiffness, are determined by its five composing layers, namely epithelium, bowman's layer, stroma, Descemet's membrane, and the endothelium. The imbalance of biomechanical homeostasis cross cornea contributes to the occurrence of corneal diseases such as KC. KC is a progressive corneal ectasia condition featured as a cone-shaped cornea with local thinning corneal stroma. Top risk factors of KC include family history, eye rubbing, eczema, asthma and allergy (40). Associated with disorganization and undulation of tissue structure, the alteration in ocular biomechanics plays an essential role in the pathogenesis of KC. Bettahar et al. (41) reported that eye rubbing is a considerable contributing factor in corneal degeneration of KC patients. Rubbing action triggers several mechanical insults, including IOP spikes, altered shear stress, and high hydrostatic tissue pressure. For instance, vigorous rubbing can skyrocket the IOP to more than 10 times of a normal resting IOP, generating more dramatic pressure strain on the cornea (7). Mechano-transducers like YAP in stromal cells and β-catenin in epithelial cells are associated with the regulation of substrate stiffness and protease production in KC (42, 43). A comprehensive understanding of the mechanobiology of corneal diseases may pave the way for new avenues for therapeutic approaches.

### Glaucoma

Glaucoma is an irreversible visual impairment disease with substantial changes in ocular biomechanical properties. The main risk factors for glaucoma include aging, elevated IOP, family history of glaucoma, and high myopia (44). The biomechanical disturbance is indispensable in the pathogenesis of glaucoma. Moreover, the glaucomatous biomechanical stress is generated by several ocular tissues (e.g., TM, iris, peripapillary sclera, and ONH), which exert direct or indirect biomechanical roles in various subtypes of glaucoma. Here, we mainly discuss the biomechanical features of glaucoma in predominant clinical subtypes including primary congenital glaucoma (PCG), primary angle-closure glaucoma (PACG), malignant glaucoma and primary open-angle glaucoma (POAG).

PCG is characterized by the abnormal anatomical structure of the TM and anterior chamber angle, thus resulting in aqueous outflow resistance and IOP elevation in infancy (45). With the progression of the disease, the affected eye may display a larger cornea or eyeball size than normal individuals, which is named “hydrophthalmos” or “buphthalmos.” Of note, these two terms involve different etiologies and clinical features. Hydrophthalmos mainly refers to the enlargement of the cornea, with or without the whole eyeball expansion. Here, we speculate that the vitreous biomechanics might be involved in the formation of hydrophthalmos. The vitreous cavity is full of intact, dense and regularly structured vitreous gel without vitreous liquefaction in infancy (46), which acts as a favorable mechanical buffer to counteract the anterior pressure derived from the elevated IOP. The posterior segment of the eye tissues is less susceptible to mechanical stimuli than the cornea, thus the primary ocular deformation occurs in the cornea. However, with the constant IOP elevation and chronic damage to the eye tissues, the biomechanical buffering role of the vitreous cannot fully offset the excessive pressure impacted on the still-elastic young eye, eventually forming “buphthalmos” featured by sclera distension and eyeball enlargement (47).

The relative pupillary block between the iris and lens is the common mechanism of PACG. The pathogenic structural changes include lens antedisplacement, plateau iris configuration and iris bombe, sequentially inducing the pupillary block accompanied by the obstruction of aqueous humor. These changes raise the pressure difference between the posterior chamber and anterior chamber, contributing to the angle closure and IOP elevation (48). Severe acute angle-closure glaucoma can lead to morphological changes of lens, known as the glaucomatous fleck, which is an irregular grayish-white spot in the anterior lens capsule at the pupillary area. It might relate to nutritional disorders, or direct contact between the iris and the anterior lens capsule under a high IOP attack (49).

Malignant glaucoma is featured with the progressive elevation of IOP and resistance to therapeutics. It is also termed as ciliary block glaucoma, vitreous displacement glaucoma, aqueous humor misdirection syndrome, or vitreociliary block glaucoma. Although the underlying etiology of malignant glaucoma is not well-elucidated, some widely-accepted theories indicate that it may result from the anterior displacement of irido-crystalline diaphragm elicited by the swelling, hypertrophy, or anterior displacement of the ciliary body, or by the laxity of zonular (50). The increasing pressure difference in these compartments blocks the normal forward passage of aqueous humor and traps the refluxed aqueous flow in the vitreous cavity. The excessive pressure difference between vitreous cavity and anterior chamber escalates the anterior displacement of irido-crystalline diaphragm, accompanied by a flattened anterior chamber (51). Recent research proposed potential risk factors such as choroidal expansion and anterior vitreous abnormalities in malignant glaucoma, subsequent confirmation still needs to be performed (52).

The prominent role of TLCPD, established by IOP and CSFP across the ONH, is well-acknowledged in the POAG etiology. Our previous studies identified that patients with normal-tension glaucoma had significantly lower CSFP and a higher TLCPD when compared with the normal subjects (53). The increased TLCPD may contribute to the LC deformation involving astrocyte migration, axonal bundle disorganization and extracellular matrix alternation (54, 55). Specifically, the individual role of these two forming ingredients is not equivalent, IOP-driven biomechanical effects display a more dominant role than CSFP (56). Multiple mechanosensitive channels such as Piezo, TRPV4, and TREK-1 are proven to have biomechanical effects in glaucoma on an experimental basis. The chemical inhibition or genetic ablation of these channels significantly ameliorates pathological phenotypes of optic nerve degeneration caused by IOP elevation, indicating the potential therapeutic roles of targeting mechanosensitive channels in glaucoma (57).

### Spaceflight-associated neuro-ocular syndrome

After a long-term spaceflight, some astronauts were bothered by visual changes associated with ocular conditions, which were termed spaceflight-associated neuro-ocular syndrome (SANS) (58). The occurrence of SANS is primarily attributed to the chronic exposure of the astronauts to the unique microgravity environment during long-term spaceflight. Other associated risk factors include radiation exposure, inflated ambient CO_2_ concentrations, high salt diets, intense resistance exercise, nutritional disturbance, and genetic variations in the one-carbon metabolism pathway (59, 60). Due to the prolonged microgravity exposure, SANS is generally characterized as fluid redistribution in the optic nerve sheath (ONS) and cerebrospinal fluid cavity (61). The cephalad fluid shifts occurring with weightlessness elevate the biomechanical strain transmitted to the ONH, as evidenced by progressive papilloedema and globe-flattening (62). To better distinguish the pathologies, a terrestrial analog called 6-degree head-down tilt bed rest (HDTBR) was established. After 30 days of examination, similar ocular changes of SANS were also identified in the HDTBR model with elevated ICP (63). Moreover, the alterations of TLCPD are also suspicious in the development of optic disc edema with increased ONS pressure protruding the LC anteriorly (64). Several countermeasures have been proposed to rebalance the biomechanical homeostasis at the site of ONH in SANS cases. A lower body negative pressure apparatus has been used to combat the cephalad fluid shift and showed a significant reduction of ICP in HDTBR testing (65). To rebuild the positive and posteriorly-directed pressure gradient, a swim goggles compression experiment was adopted to increase IOP and restore the normal TLCPD (64). These discoveries highlight the malignant impacts of imbalanced TLCPD induced by idiopathic intracranial hypertension, underscoring the fundamental role of biomechanical homeostasis in ocular health.

### Retinal vein occlusion

Retinal vein occlusion (RVO) is a constellation of hypertensive retinopathies associated with multiple risk factors like aging, systemic hypertension, cardiovascular disorders, hyperlipidemia, diabetes, glaucoma, and thrombophilic mutations (e.g., antithrombin, protein C or protein S) (66, 67). The physical obstruction of the retinal venous system is generally induced by thrombosis, deformation of the vein wall, and external biomechanical compression secondary to glaucoma (68). Mechanically, it is postulated that the elevated IOP compresses the LC and optic disc, thereby leading to the stretching and weakening of the vessel wall, which further predisposes the retinal vein to occlusion (69). An excessive dropout of parapapillary choroidal microvasculature is also observed in RVO patients (70). Meanwhile, the direct biomechanical insult of IOP obstructs the retinal vein drainage and induces venous stasis, consequently exacerbating the intimal proliferation in the vein (71). Substantial evidence is required to further elucidate the underlying biomechanical changes in RVO etiology.

### Myopia

As the most common refractive condition, myopia often starts in childhood and is manifested as short- or near-sightedness. Emerging evidence has supported the role of nature (genetics and inheritance) and nurture (environment and lifestyle) in the onset of myopia (72). Specifically, the major risk factors include higher education levels, prolonged near-work time, reduced outdoor activities, and inherited genetic predispositions (e.g., MYP1 family, ZNF644, SCO2, BSG, APLP2, etc.) (72–74). The biological deformation of myopia is typically characterized by an elongated posterior scleral shell. Severe scleral thinning in high myopia can lead to the biomechanical deformation of the posterior scleral wall, manifested as posterior staphyloma. It has been validated in several animal studies that the sclera thins during experimental myopia, suggesting the distinct role of scleral remodeling in the pathological axial elongation (75, 76). Scleral remodeling is a process of micro-deformation in a volume-conserving pattern, which results in the rearrangement of existing tissue materials. In highly myopic eyes, this mechanical adaption to the scleral tension is even greater than an equivalent IOP attack in the aspect of globe enlargement and posterior thinning of the eye wall (77). Besides, David et al. (78) have studied the impact of vitreous torsional oscillation stress on the retina secondary to regular ocular motion. They found that the high myopia eye is hypersensitive to this chronic mechanical torsional stress, speculating it as the underlying cause of rupture-induced retinal detachment occurred in pathological myopia.

## Discussion

Emerging evidence has associated biomechanical homeostasis with ocular health. The biomechanical features of the anterior segment (cornea, sclera, drainage route, and lens capsule) and the posterior segment (vitreous, Bruch's membrane, choroid, retina, and optic nerve) of the eye have been documented with substantial evidence, whereas the understanding of inner homeostasis between different tissues remained unclear. Knowledge of these physical interactions is pivotal not only to clarify the underlying pathogenesis of a vast range of retinal and vitreoretinal diseases, such as DR, KC and glaucoma, but also to optimize the surgical handling of ocular tissues and the design of novel therapies.

Till now, the present studies of biomechanical analysis mainly focus on glaucoma (79), DR (80) and high myopia (81). The mainstream analytical methods of ocular biomechanics can be summarized into three subcategories, including (1) computational modeling (e.g., finite element modeling): a simplified model under ideal conditions with substantial variations from real-life situations (79); (2) microfluidic eye chips: a newly emerging 3D cell culture system providing novel insights for biomechanical studies *in vitro* (82); (3) the commercially available medical equipment (e.g., Corvis ST and wearable IOP biosensor) for clinical assessment (83, 84).

Novel treatment approaches and concepts have been proposed for the restoration of biomechanical homeostasis in ocular disorders. For the anterior segment, corneal cross-linking is widely utilized to increase corneal biomechanical resistance in treating ectasia and KC (85). Similarly, as collagen fiber crimping and re-alignment are observed in the development of myopia, collagen crosslinking has also been recommended as a potential therapeutic strategy for progressive myopia (86). For the posterior segment, LC stiffening is a common pathology in multiple ocular diseases like glaucoma and DR, which can be triggered by elevated IOP and increased AGEs, respectively. To alleviate the stresses and strains, collagenase treatment has been investigated in human cadaver eyes for the reduction of the biomechanical stiffness of LC (87). Besides, the posterior segment ring implantation (e.g., intrascleral or subarachnoid space ring) has been proposed as a potential countermeasure to delay the LC deformation in glaucoma at the conceptional level (12).

Altogether, biomechanical homeostasis is crucial to maintain the physiological function of the eye. In-depth acknowledgment of ocular biomechanics could help us better understand the underlying mechanical properties and molecular mechanisms in different ophthalmic conditions, further providing novel diagnostic methods and countermeasures from the perspective of mechanobiology.

## Author contributions

The topic was devised and conceptualized by NW. YC and TR conducted the literature review and wrote the manuscript. All authors have read and agreed to the published final version.

## References

[1] Giri B Dey S Das T Sarkar M Banerjee J Dash SK. Chronic hyperglycemia mediated physiological alteration and metabolic distortion leads to organ dysfunction, infection, cancer progression and other pathophysiological consequences: an update on glucose toxicity. Biomed Pharmacother. (2018) 107:306–28. 10.1016/j.biopha.2018.07.157 30098549

[2] Tang WH Martin KA Hwa J. Aldose reductase, oxidative stress, and diabetic mellitus. Front Pharmacol. (2012) 3:87. 10.3389/fphar.2012.00087 22582044 PMC3348620

[3] Cohen E Kramer M Shochat T Goldberg E Krause I. Relationship between serum glucose levels and intraocular pressure, a population-based cross-sectional study. J Glaucoma. (2017) 26:652–6. 10.1097/IJG.0000000000000700 28598960

[4] Voutilainen-Kaunisto R Niskanen L Uusitupa M Teräsvirta M. Iris transluminance in type 2 diabetes. Acta Ophthalmol Scand. (2002) 80:64–8. 10.1034/j.1600-0420.2002.800113.x 11906307

[5] Hou H Shoji T Zangwill LM Moghimi S Saunders LJ Hasenstab K. Progression of primary open-angle glaucoma in diabetic and nondiabetic patients. Am J Ophthalmol. (2018) 189:1–9. 10.1016/j.ajo.2018.02.002 29447914 PMC5916320

[6] Hatze H. Letter: The meaning of the term “biomechanics”. J Biomech. (1974) 7:189–90. 10.1016/0021-9290(74)90060-8 4837555

[7] Yang S Zhang J Tan Y Wang Y. Unraveling the mechanobiology of cornea: from bench side to the clinic. Front Bioeng Biotechnol. (2022) 10:953590. 10.3389/fbioe.2022.953590 36263359 PMC9573972

[8] Shaw AJ Collins MJ Davis BA Carney LG. Eyelid pressure and contact with the ocular surface. Invest Ophthalmol Vis Sci. (2010) 51:1911–7. 10.1167/iovs.09-4090 19834035

[9] Jones MB Fulford GR Please CP McElwain DL Collins MJ. Elastohydrodynamics of the eyelid wiper. Bull Math Biol. (2008) 70:323–43. 10.1007/s11538-007-9252-7 18066629

[10] Qin Z Meng L Yang F Zhang C Wen B. Aqueous humor dynamics in human eye: a lattice Boltzmann study. Math Biosci Eng. (2021) 18:5006–28. 10.3934/mbe.2021255 34517475

[11] Masterton S Ahearne M. Mechanobiology of the corneal epithelium. Exp Eye Res. (2018) 177:122–9. 10.1016/j.exer.2018.08.001 30086260 PMC6280025

[12] Boote C Sigal IA Grytz R Hua Y Nguyen TD Girard MJA. Scleral structure and biomechanics. Prog Retin Eye Res. (2020) 74:100773. 10.1016/j.preteyeres.2019.100773 31412277 PMC7187923

[13] Stamer WD Braakman ST Zhou EH Ethier CR Fredberg JJ Overby DR. Biomechanics of Schlemm's canal endothelium and intraocular pressure reduction. Prog Retin Eye Res. (2015) 44:86–98. 10.1016/j.preteyeres.2014.08.002 25223880 PMC4268318

[14] Sherwood JM Stamer WD Overby DR A. model of the oscillatory mechanical forces in the conventional outflow pathway. J R Soc Interface. (2019) 16:20180652. 10.1098/rsif.2018.0652 30958169 PMC6364644

[15] Huang D Xu C Guo R Ji J Liu W. Anterior lens capsule: biomechanical properties and biomedical engineering perspectives. Acta Ophthalmol. (2021) 99:e302–9. 10.1111/aos.14600 32914585

[16] Krag S Andreassen TT. Mechanical properties of the human posterior lens capsule. Invest Ophthalmol Vis Sci. (2003) 44:691–6. 10.1167/iovs.02-0096 12556400

[17] Silver DM Quigley HA. Aqueous flow through the iris-lens channel: estimates of differential pressure between the anterior and posterior chambers. J Glaucoma. (2004) 13:100–7. 10.1097/00061198-200404000-00004 15097254

[18] Di Michele F Tatone A Romano MR Repetto R. A mechanical model of posterior vitreous detachment and generation of vitreoretinal tractions. Biomech Model Mechanobiol. (2020) 19:2627–41. 10.1007/s10237-020-01360-1 32642790

[19] Tram NK Swindle-Reilly KE. Rheological properties and age-related changes of the human vitreous humor. Front Bioeng Biotechnol. (2018) 6:199. 10.3389/fbioe.2018.00199 30619846 PMC6305337

[20] Meskauskas J Repetto R Siggers JH. Shape change of the vitreous chamber influences retinal detachment and reattachment processes: is mechanical stress during eye rotations a factor? Invest Ophthalmol Vis Sci. (2012) 53:6271–81. 10.1167/iovs.11-9390 22899755 PMC3465013

[21] Ugarte M Hussain AA Marshall J. An experimental study of the elastic properties of the human Bruch's membrane-choroid complex: relevance to ageing. Br J Ophthalmol. (2006) 90:621–6. 10.1136/bjo.2005.086579 16622094 PMC1857059

[22] Ferrara M Lugano G Sandinha MT Kearns VR Geraghty B Steel DHW. Biomechanical properties of retina and choroid: a comprehensive review of techniques and translational relevance. Eye. (2021) 35:1818–32. 10.1038/s41433-021-01437-w 33649576 PMC8225810

[23] Liu KC Fleischman D Lee AG Killer HE Chen JJ Bhatti MT. Current concepts of cerebrospinal fluid dynamics and the translaminar cribrosa pressure gradient: a paradigm of optic disk disease. Surv Ophthalmol. (2020) 65:48–66. 10.1016/j.survophthal.2019.08.005 31449832

[24] Ho CH Wong JKW. Role of 24-hour intraocular pressure monitoring in glaucoma management. J Ophthalmol. (2019) 2019:3632197. 10.1155/2019/3632197 31641532 PMC6770303

[25] van den Bosch J Pennisi V Invernizzi A Mansouri K Weinreb RN Thieme H. Implanted microsensor continuous IOP telemetry suggests gaze and eyelid closure effects on IOP-A preliminary study. Invest Ophthalmol Vis Sci. (2021) 62:8. 10.1167/iovs.62.6.8 33956052 PMC8107486

[26] Mansouri K Gillmann K Rao HL Weinreb RN. Weekly and seasonal changes of intraocular pressure measured with an implanted intraocular telemetry sensor. Br J Ophthalmol. (2021) 105:387–91. 10.1136/bjophthalmol-2020-315970 32499329

[27] van den Bosch J Pennisi V Mansouri K Weinreb RN Thieme H Hoffmann MB. Effect of eyelid muscle action and rubbing on telemetrically obtained intraocular pressure in patients with glaucoma with an IOP sensor implant. Br J Ophthalmol. (2022). 10.1136/bjophthalmol-2021-320508 35701079 PMC10579178

[28] Schecterson LC Pazevic AA Yang R Matulef K Gordon SE. TRPV1, TRPA1, and TRPM8 are expressed in axon terminals in the cornea: TRPV1 axons contain CGRP and secretogranin II; TRPA1 axons contain secretogranin 3. Mol Vis. (2020) 26:576–87. 32863706 PMC7438417

[29] Tran VT Ho PT Cabrera L Torres JE Bhattacharya SK. Mechanotransduction channels of the trabecular meshwork. Curr Eye Res. (2014) 39:291–303. 10.3109/02713683.2013.842593 24215462

[30] KriŽaj D Cordeiro S Strauß O. Retinal TRP channels: Cell-type-specific regulators of retinal homeostasis and multimodal integration. Prog Retin Eye Res. (2022) 23:101114. 10.1016/j.preteyeres.2022.101114 36163161 PMC9897210

[31] Giblin JP Comes N Strauss O Gasull X. Ion channels in the eye: involvement in ocular pathologies. Adv Protein Chem Struct Biol. (2016) 104:157–231. 10.1016/bs.apcsb.2015.11.006 27038375

[32] Lin KY Hsih WH Lin YB Wen CY Chang TJ. Update in the epidemiology, risk factors, screening, and treatment of diabetic retinopathy. J Diabetes Investig. (2021) 12:1322–5. 10.1111/jdi.13480 33316144 PMC8354492

[33] Li Y Mitchell W Elze T Zebardast N. Association between diabetes, diabetic retinopathy, and glaucoma. Curr Diab Rep. (2021) 21:38. 10.1007/s11892-021-01404-5 34495413

[34] Abikoye TM Oluleye TS Aribaba OT Musa KO Idowu OO Onakoya AO. Is primary open-angle glaucoma a risk factor for diabetic retinopathy? Int Ophthalmol. (2020) 40:3233–40. 10.1007/s10792-020-01507-0 32696101

[35] Ren R Wang N Zhang X Tian G Jonas JB. Cerebrospinal fluid pressure correlated with body mass index. Graefes Arch Clin Exp Ophthalmol. (2012) 250:445–6. 10.1007/s00417-011-1746-1 21814821

[36] Flammer J Konieczka K. Retinal venous pressure: the role of endothelin. EPMA J. (2015) 6:21. 10.1186/s13167-015-0043-1 26504500 PMC4620652

[37] Orduña Ríos M Noguez Imm R Hernández Godínez NM Bautista Cortes AM López Escalante DD Liedtke W. TRPV4 inhibition prevents increased water diffusion and blood-retina barrier breakdown in the retina of streptozotocin-induced diabetic mice. PLoS One. (2019) 14:e0212158. 10.1371/journal.pone.0212158 31048895 PMC6497373

[38] Guarino BD Paruchuri S Thodeti CK. The role of TRPV4 channels in ocular function and pathologies. Exp Eye Res. (2020) 201:108257. 10.1016/j.exer.2020.108257 32979394 PMC7736234

[39] McKay TB Priyadarsini S Karamichos D. Mechanisms of collagen crosslinking in diabetes and keratoconus. Cells. (2019) 8:1239. 10.3390/cells8101239 31614631 PMC6830090

[40] Santodomingo-Rubido J Carracedo G Suzaki A Villa-Collar C Vincent SJ Wolffsohn JS. Keratoconus: an updated review. Cont Lens Anterior Eye. (2022) 45:101559. 10.1016/j.clae.2021.101559 34991971

[41] Bettahar T Rahmoune C Benazzouz D. Keratoconus prognosis study for patients with corneal external mechanical stress mode. Int Ophthalmol. (2020) 40:1673–86. 10.1007/s10792-020-01335-2 32219616

[42] Amit C Padmanabhan P Narayanan J. Deciphering the mechanoresponsive role of β-catenin in keratoconus epithelium. Sci Rep. (2020) 10:21382. 10.1038/s41598-020-77138-3 33288782 PMC7721701

[43] Dou S Wang Q Zhang B Wei C Wang H Liu T. Single-cell atlas of keratoconus corneas revealed aberrant transcriptional signatures and implicated mechanical stretch as a trigger for keratoconus pathogenesis. Cell Discov. (2022) 8:66. 10.1038/s41421-022-00452-9 35821117 PMC9276680

[44] Schuster AK Erb C Hoffmann EM Dietlein T Pfeiffer N. The diagnosis and treatment of glaucoma. Dtsch Arztebl Int. (2020) 117:225–34. 10.3238/arztebl.2020.0225 32343668 PMC7196841

[45] Tehreem R Arooj A Siddiqui SN Naz S Afshan K Firasat S. Mutation screening of the CYP1B1 gene reveals thirteen novel disease-causing variants in consanguineous Pakistani families causing primary congenital glaucoma. PLoS ONE. (2022) 17:e0274335. 10.1371/journal.pone.0274335 36083974 PMC9462810

[46] Holekamp NM. The vitreous gel: more than meets the eye. Am J Ophthalmol. (2010) 149:32–6. 10.1016/j.ajo.2009.07.036 19875090

[47] Feroze KB Blair K Patel BC. Buphthalmos. StatPearls. Treasure Island, FL: StatPearls Publishing Copyright © 2022, StatPearls Publishing LLC (2022).

[48] Gupta B Angmo D Yadav S Dada T Gupta V Sihota R. Quantification of iridotrabecular contact in primary angle-closure disease. J Glaucoma. (2020) 29:681–8. 10.1097/IJG.0000000000001572 32555058

[49] Soh ZD Thakur S Majithia S Nongpiur ME Cheng CY. Iris and its relevance to angle closure disease: a review. Br J Ophthalmol. (2021) 105:3–8. 10.1136/bjophthalmol-2020-316075 32193222

[50] Dorado-López-Rosado AM Mencía-Gutiérrez E Polo-García M Gutiérrez-Díaz E. Vitreociliary block in a patient with uveitis and previous laser posterior capsulotomy. Cureus. (2021) 13:e14786. 10.7759/cureus.14786 34094748 PMC8169092

[51] Grzybowski A Kanclerz P. Acute and chronic fluid misdirection syndrome: pathophysiology and treatment. Graefes Arch Clin Exp Ophthalmol. (2018) 256:135–54. 10.1007/s00417-017-3837-0 29110086 PMC5748435

[52] Kaplowitz K Yung E Flynn R Tsai JC. Current concepts in the treatment of vitreous block, also known as aqueous misdirection. Surv Ophthalmol. (2015) 60:229–41. 10.1016/j.survophthal.2014.12.004 25639795

[53] Ren R Jonas JB Tian G Zhen Y Ma K Li S. Cerebrospinal fluid pressure in glaucoma: a prospective study. Ophthalmology. (2010) 117:259–66. 10.1016/j.ophtha.2009.06.058 19969367

[54] Lee EJ Han JC Park DY Kee C. A neuroglia-based interpretation of glaucomatous neuroretinal rim thinning in the optic nerve head. Prog Retin Eye Res. (2020) 77:100840. 10.1016/j.preteyeres.2020.100840 31982595

[55] Cheng Y Wu S Yan X Liu Q Lin D Zhang J Wang N. Human Pro370Leu mutant myocilin induces the phenotype of open-angle glaucoma in transgenic mice. Cell Mol Neurobiol. (2022). 10.1007/s10571-022-01280-x 36069958 PMC11412175

[56] Karimi A Razaghi R Rahmati SM Girkin CA Downs JC. Relative contributions of intraocular and cerebrospinal fluid pressures to the biomechanics of the lamina cribrosa and laminar neural tissues. Invest Ophthalmol Vis Sci. (2022) 63:14. 10.1167/iovs.63.11.14 36255364 PMC9587471

[57] KriŽaj D. What is glaucoma? In:Kolb H Fernandez E Nelson R, editors. Webvision: The Organization of the Retina and Visual System. Salt Lake City, UT: University of Utah Health Sciences Center Copyright: © 2022 Webvision (1995). 21413389

[58] Wojcik P Kini A Al Othman B Galdamez LA Lee AG. Spaceflight associated neuro-ocular syndrome. Curr Opin Neurol. (2020) 33:62–7. 10.1097/WCO.0000000000000778 31789708

[59] Smith SM Zwart SR Heer M. Human Adaptation to Space Flight: The Role of Nutrition. Houston, TX: National Aeronautics and Space Administration, Lyndon B Johnson Space Center (2014).

[60] Zwart SR Gibson CR Mader TH Ericson K Ploutz-Snyder R Heer M. Vision changes after spaceflight are related to alterations in folate- and vitamin B-12-dependent one-carbon metabolism. J Nutr. (2012) 142:427–31. 10.3945/jn.111.154245 22298570

[61] Shinojima A. Possible factors associated with spaceflight-associated neuro-ocular syndrome. JAMA Ophthalmol. (2020) 138:172–3. 10.1001/jamaophthalmol.2019.5365 31876941

[62] Roberts DR Petersen LG. Studies of hydrocephalus associated with long-term spaceflight may provide new insights into cerebrospinal fluid flow dynamics here on earth. JAMA Neurol. (2019) 76:391–2. 10.1001/jamaneurol.2018.4891 30673794

[63] Laurie SS Macias BR Dunn JT Young M Stern C Lee SMC. Optic disc edema after 30 days of strict head-down tilt bed rest. Ophthalmology. (2019) 126:467–8. 10.1016/j.ophtha.2018.09.042 30308219

[64] Scott JM Tucker WJ Martin D Crowell JB Goetchius E Ozgur O. Association of exercise and swimming goggles with modulation of cerebro-ocular hemodynamics and pressures in a model of spaceflight-associated neuro-ocular syndrome. JAMA Ophthalmol. (2019) 137:652–9. 10.1001/jamaophthalmol.2019.0459 30998818 PMC6567831

[65] Petersen LG Lawley JS Lilja-Cyron A Petersen JCG Howden EJ Sarma S. Lower body negative pressure to safely reduce intracranial pressure. J Physiol. (2019) 597:237–48. 10.1113/JP276557 30286250 PMC6312426

[66] Song P Xu Y Zha M Zhang Y Rudan I. Global epidemiology of retinal vein occlusion: a systematic review and meta-analysis of prevalence, incidence, and risk factors. J Glob Health. (2019) 9:010427. 10.7189/jogh.09.010427 31131101 PMC6513508

[67] Bucciarelli P Passamonti SM Gianniello F Artoni A Martinelli I. Thrombophilic and cardiovascular risk factors for retinal vein occlusion. Eur J Intern Med. (2017) 44:44–8. 10.1016/j.ejim.2017.06.022 28684050

[68] Khayat M Williams M Lois N. Ischemic retinal vein occlusion: characterizing the more severe spectrum of retinal vein occlusion. Surv Ophthalmol. (2018) 63:816–50. 10.1016/j.survophthal.2018.04.005 29705175

[69] Kim YN Shin JW Park YJ Lee JY Kim JG Yoon YH. Glaucoma as a prognostic factor of central retinal vein occlusion: visual and anatomical outcomes and occurrence of ischaemic central retinal vein occlusion. Acta Ophthalmol. (2021) 99:e523–30. 10.1111/aos.14608 33113286

[70] Baek J Park HL Kim SA Hong KE Jeon SJ Shin DY. Parapapillary choroidal microvasculature dropout in branched retinal vein occlusion and glaucoma. Invest Ophthalmol Vis Sci. (2022) 63:27. 10.1167/iovs.63.3.27 35348587 PMC8976936

[71] Hayreh SS Zimmerman MB Beri M Podhajsky P. Intraocular pressure abnormalities associated with central and hemicentral retinal vein occlusion. Ophthalmology. (2004) 111:133–41. 10.1016/j.ophtha.2003.03.002 14711725

[72] Baird PN Saw SM Lanca C Guggenheim JA Smith EL Zhou X. Myopia. Nat Rev Dis Primers. (2020) 6:99. 10.1038/s41572-020-00231-4 33328468

[73] Li SM Ran AR Kang MT Yang X Ren MY Wei SF. Effect of text messaging parents of school-aged children on outdoor time to control myopia: a randomized clinical trial. JAMA Pediatr. (2022) 176:1077–83. 10.1001/jamapediatrics.2022.3542 36155742 PMC9513710

[74] Cai XB Shen SR Chen DF Zhang Q Jin ZB. An overview of myopia genetics. Exp Eye Res. (2019) 188:107778. 10.1016/j.exer.2019.107778 31472110

[75] McBrien NA Cornell LM Gentle A. Structural and ultrastructural changes to the sclera in a mammalian model of high myopia. Invest Ophthalmol Vis Sci. (2001) 42:2179–87. 11527928

[76] Norton TT Rada JA. Reduced extracellular matrix in mammalian sclera with induced myopia. Vision Res. (1995) 35:1271–81. 10.1016/0042-6989(94)00243-F 7610587

[77] Markov PP Eliasy A Pijanka JK Htoon HM Paterson NG Sorensen T. Bulk changes in posterior scleral collagen microstructure in human high myopia. Mol Vis. (2018) 24:818–33. 30713421 PMC6334987

[78] David T Smye S Dabbs T James T. A model for the fluid motion of vitreous humour of the human eye during saccadic movement. Phys Med Biol. (1998) 43:1385–99. 10.1088/0031-9155/43/6/001 9651012

[79] Mao Y Yang D Li J Liu J Hou R Zhang Z. Finite element analysis of trans-lamina cribrosa pressure difference on optic nerve head biomechanics: the Beijing Intracranial and Intraocular Pressure Study. Sci China Life Sci. (2020) 63:1887–94. 10.1007/s11427-018-1585-8 32447541

[80] Rebhan J Parker LP Kelsey LJ Chen FK Doyle BJ. A computational framework to investigate retinal haemodynamics and tissue stress. Biomech Model Mechanobiol. (2019) 18:1745–57. 10.1007/s10237-019-01172-y 31140054

[81] Sedaghat MR Momeni-Moghaddam H Azimi A Fakhimi Z Ziaei M Danesh Z. Corneal biomechanical properties in varying severities of myopia. Front Bioeng Biotechnol. (2020) 8:595330. 10.3389/fbioe.2020.595330 33553113 PMC7859342

[82] Zhao Y Hu G Yan Y Wang Z Liu X Shi H. Biomechanical analysis of ocular diseases and its in vitro study methods. Biomed Eng Online. (2022) 21:49. 10.1186/s12938-022-01019-1 35870978 PMC9308301

[83] Nakao Y Kiuchi Y Okumichi H. Evaluation of biomechanically corrected intraocular pressure using Corvis ST and comparison of the Corvis ST, noncontact tonometer, and Goldmann applanation tonometer in patients with glaucoma. PLoS ONE. (2020) 15:e0238395. 10.1371/journal.pone.0238395 32966284 PMC7510959

[84] Araci IE Agaoglu S Lee JY Rivas Yepes L Diep P Martini M. Flow stabilization in wearable microfluidic sensors enables noise suppression. Lab Chip. (2019) 19:3899–908. 10.1039/C9LC00842J 31641709

[85] Ma J Wang Y Wei P Jhanji V. Biomechanics and structure of the cornea: implications and association with corneal disorders. Surv Ophthalmol. (2018) 63:851–61. 10.1016/j.survophthal.2018.05.004 29857022

[86] Wang M Corpuz CCC Zhang F. Shaping eyeballs by scleral collagen cross-linking: a hypothesis for myopia treatment. Front Med. (2021) 8:655822. 10.3389/fmed.2021.655822 34277654 PMC8282923

[87] Spoerl E Boehm AG Pillunat LE. The influence of various substances on the biomechanical behavior of lamina cribrosa and peripapillary sclera. Invest Ophthalmol Vis Sci. (2005) 46:1286–90. 10.1167/iovs.04-0978 15790892

